# Patient, Physician, and Caregiver Preferences for Lung Cancer Treatment: A Systematic Review of Discrete Choice Experiments

**DOI:** 10.3390/healthcare14050584

**Published:** 2026-02-26

**Authors:** Sida Wang, Yun Liu, Mengyu Yang, Linning Wang, Jie Yu, Xiaoxi Xie, Feng Chang, Yun Lu

**Affiliations:** School of International Pharmaceutical Business, China Pharmaceutical University, No. 639 Longmian Avenue, Nanjing 211198, China

**Keywords:** lung cancer, discrete choice experiments, different stakeholders, systematic review, preferences

## Abstract

**Objectives:** This systematic review aimed to synthesize evidence from discrete choice experiments to explore the preferences of patients, physicians, and caregivers regarding lung cancer treatment. **Methods:** A systematic literature search was conducted utilizing the PubMed, Web of Science, Embase, and Scopus databases, encompassing publications up to 12 July 2024. We included published discrete choice experiment (DCE) studies that assessed preferences for lung cancer treatment among patients, physicians, or caregivers, with no restrictions on country, language, publication date, or disease stage. Two researchers independently conducted the literature screening and data extraction. The included studies were assessed for quality using the PREFS checklist. **Results:** Among the 1086 studies identified, 18 studies met the eligibility criteria. A total of 115 attributes were extracted and categorized into three main categories: outcome, process, and cost, with subcategories under each. Regarding the relative importance of attributes, heterogeneity was observed among stakeholders. The PREFS scores of the 18 included studies, with an average score of 3.8, reflect the high overall quality of these studies. **Conclusions:** This review revealed both commonalities and differences in lung cancer treatment preferences among patients, physicians, and caregivers. However, existing studies have certain limitations in the coverage of study populations, the scope of attributes, and the research design of the experiments.

## 1. Introduction

Cancer presents a major global public health concern and remains one of the leading causes of mortality and disease burden worldwide [1]. According to the latest global cancer burden data from the International Agency for Research on Cancer (IARC) of the World Health Organization (WHO), an estimated 20 million new cancer cases and 9.7 million cancer deaths occurred globally in 2022 [2]. With 2.5 million new cases and 1.8 million deaths [2], lung cancer stands as the cancer type with the highest incidence and mortality rates globally, imposing substantial psychological and economic burdens not only on patients but also on their families and society. Lung cancer is classified into two main types based on pathological characteristics: non-small cell lung cancer (NSCLC) and small cell lung cancer (SCLC), with NSCLC being the most prevalent, accounting for approximately 80% to 85% of all lung cancer cases [3,4]. With continuous advancements in medical technology, treatment options for lung cancer have expanded to encompass not only traditional methods such as surgery, chemotherapy, and radiotherapy, but also various emerging therapies like molecular targeted therapy, photothermal therapy, and immunotherapy [5,6,7]. However, this diversity may increase the complexity of treatment decisions, requiring a comprehensive assessment of various factors, including the patient’s clinical condition, treatment expectations, quality of life, and potential side effects. In this context, patients, physicians, and caregivers (the latter used here as an umbrella term for individuals involved in supporting patients and/or participating in treatment decisions) may exhibit diverse preferences when selecting treatment options. Physicians rely on their expertise to formulate a rational and personalized treatment strategy for the patient. Unlike patients, caregivers do not directly experience treatment benefits and harms, but their preferences may reflect caregiving burden and practical considerations. Meanwhile, patients and caregivers need to weigh the different treatment options in alignment with their actual condition and expectations regarding quality of life to select the most appropriate treatment. Therefore, it is essential to conduct an in-depth exploration of the differences in treatment preferences among patients, physicians, and caregivers in the management of lung cancer.

Preferences can be categorized into 2 types based on the method of measurement: revealed preferences and stated preferences. Specifically, revealed preferences involve analyzing actual observed behaviors, reflecting individuals’ authentic decision-making processes. In contrast, stated preferences are based on feedback collected through surveys to assess respondents’ preferences for specific options or scenarios under hypothetical conditions [8,9]. In recent years, Discrete Choice Experiments (DCE) have become a popular tool for assessing stated preferences and have been widely used in the field of health economics [10,11,12], increasingly to quantify the preferences of patients, physicians, and other stakeholders [13]. In DCE, a series of choice scenarios simulating real-life decision-making contexts is designed by combining different attributes and levels, prompting respondents to select their most favored option from two or more alternatives. Subsequently, appropriate statistical models are employed to quantitatively analyze respondents’ choices and estimate their underlying preferences for various attributes and levels [13,14]. DCEs infer preferences by analyzing respondents’ choice behaviors. The choice scenarios closely resemble real-world decision-making contexts, enabling reliable preference measurement in practical applications. In contrast, simple ratings/rankings or qualitative interviews alone are less suited to producing directly comparable trade-off estimates. Therefore, to comprehensively understand the preferences of different stakeholders regarding lung cancer treatment, it is essential to review and analyze existing DCE studies systematically.

Previous systematic reviews on preferences for lung cancer treatment have primarily focused on the patient’s perspective [15,16]. However, as treatment decisions involve multiple stakeholders, relying solely on patient preferences fails to capture the complexity of real-world clinical decisions and may overlook critical barriers to implementation. Despite numerous studies exploring the preferences of physicians and caregivers in lung cancer treatment, the differences in preferences between them and patients are still lacking. Therefore, this study aims to conduct a systematic review of DCEs and compare the preferences of patients, physicians, and caregivers regarding lung cancer treatment. The findings of this study will not only deepen the understanding of the decision-making process in lung cancer treatment among various stakeholders but also provide vital insights for personalized lung cancer treatment planning.

## 2. Methods

This systematic review was conducted in accordance with the Preferred Reporting Items for Systematic Reviews and Meta-Analyses (PRISMA) guidelines established by Moher et al. [17]. The study protocol was registered with PROSPERO (CRD42024623604). No deviations from the registered protocol occurred.

### 2.1. Search Strategy

The literature search for this systematic review was conducted across four relevant databases: PubMed, Web of Science, Embase, and Scopus. The search timeframe covered the period from each database’s inception to 12 July 2024. The search terms included two categories: those related to discrete choice experiments (DCEs) and those related to lung cancer. The DCE-related search terms were derived from previous systematic reviews on DCEs, including terms such as “conjoint experiment”, “conjoint analysis”, “conjoint measurement”, “conjoint study”, “paired comparison”, “discrete choice”, and “stated preference” [11,12,18]. The lung cancer-related search terms were formulated using a combination of MeSH terms and free-text terms. The complete electronic search strategies for all databases were provided in Supplementary Materials Table S1.

### 2.2. Eligibility Criteria

The review question was structured using the PICO framework: patients with lung cancer and other key stakeholders involved in treatment decisions (patients, physicians, and caregivers) (P); preferences elicited in DCEs comparing alternative profiles defined by attributes and levels (I/C); and stakeholder preferences such as attribute importance or reported preference weights (O). Studies were included if they employed a DCE design to investigate the preferences of patients, physicians, or caregivers regarding lung cancer treatment. No restrictions were applied to country, language, publication date, or disease stage.

Studies meeting any of the following criteria were excluded: (1) studies not using a DCE design; (2) studies unrelated to the topic of this review; (3) studies that did not measure preferences; (4) studies assessing preferences for multiple cancer treatments simultaneously; (5) duplicate publications; (6) studies for which full-text information was unavailable; and (7) reviews, commentaries, reports, conference abstracts, dissertations, books, or other forms of grey literature. We excluded grey literature to focus on peer-reviewed DCE studies for consistent extraction and analysis.

### 2.3. Study Identification and Selection

The identification and selection of studies were conducted in stages by two researchers (S.D.W. and M.Y.Y.). First, the search results from each database were imported into the reference management software NoteExpress (version 4.1.0.10030), and duplicates were removed. Second, the titles and abstracts of the studies were preliminarily screened based on pre-defined eligibility criteria. Finally, the full texts of studies identified during the initial screening were evaluated to confirm their eligibility for inclusion. If a potentially eligible study was not in English, it would be translated for eligibility assessment and data extraction. At each stage, the screening results of the two researchers were compared, and any discrepancies or disagreements during the study identification and selection process were resolved through discussion and consultation with a third researcher (Y.L.).

### 2.4. Data Extraction and Analysis

Data extraction was independently conducted by two researchers (M.Y.Y. and Y.L.), followed by collation and verification by a third researcher (S.D.W.). The extracted information primarily included the following aspects: (1) basic information of the included studies: first author, publication year, country/region of the study, disease condition, treatment type, respondent type, sample size, response rate, and survey administration method; (2) DCE choice task design: the process of determining attributes and levels, descriptions and number of attributes, descriptions and number of levels, experimental design software, number of choice sets, use of block designs, number of choice tasks answered per respondent, number of alternatives, use of labeled designs, inclusion of opt-out/status quo options, and whether a pilot study was conducted; (3) DCE statistical analysis: econometric models used, types of variable coding, data analysis software, and common metrics for comparing relative attribute effects; (4) relative attribute importance (RAI) (extracted as reported; when not explicitly reported, attributes were ranked within each study based on the coefficient range for each attribute); (5) study conclusions and recommendations.

### 2.5. Quality Assessment

This study used the PREFS checklist developed by Joy et al. to assess the quality of the included studies [19]. The PREFS checklist is concise, easy to use, and widely applied for quality assessment in DCE studies [20,21,22,23,24,25]. It focuses on five key aspects: purpose, respondents, explanation, findings, and significance. These aspects correspond to the core reporting elements needed to interpret DCE methods and results. Each aspect is scored dichotomously, with a score of 1 if the criterion is met and 0 otherwise. The total score for each study is the sum of scores across these five aspects, ranging from 0 to 5. The primary quality assessment process was independently conducted by two researchers (L.N.W. and M.Y.Y.) using the checklist. Any unresolved disagreements were addressed through discussion or consultation with a third researcher (Y.L.). However, PREFS does not fully capture DCE-specific methodological features (e.g., attribute development, experimental design efficiency, or econometric models). Therefore, PREFS scores were interpreted as an indicator of reporting quality rather than a formal risk of bias assessment.

## 3. Results

### 3.1. Search Results

**Figure 1:**
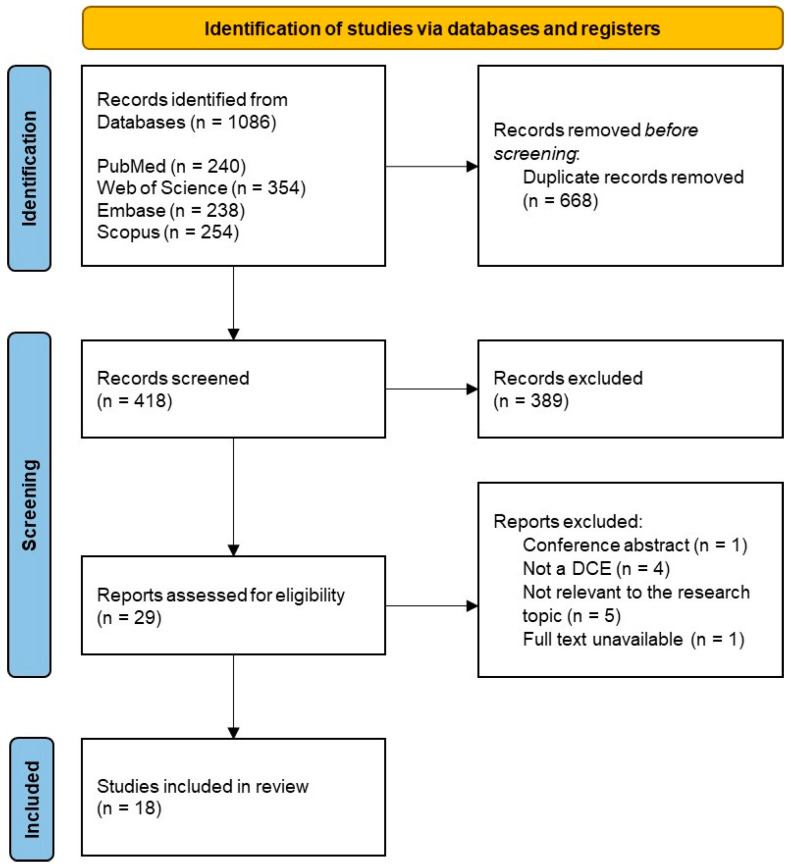
Flow of study selection through different phases of the systematic review according to the PRISMA guidelines.

Through the electronic database search, a total of 1086 records were retrieved, with 668 duplicate records removed. Of the remaining 418 records, 389 were excluded after title and abstract screening for not meeting the eligibility criteria. Consequently, 29 records were selected for full-text screening. After full-text screening, 18 studies met the inclusion criteria and were included in this systematic review [26,27,28,29,30,31,32,33,34,35,36,37,38,39,40,41,42,43]. Among the 11 full-text studies excluded, the main reasons were ineligible study design (n = 9) and inability to extract relevant data (n = 2). The study selection process is illustrated in Figure 1, following the PRISMA guidelines.

### 3.2. General Characteristics of Included Studies

Table 1 summarizes the key characteristics of the included studies. These studies were published between 2012 and 2024, with 14 studies (78%) published after 2020 [30,31,32,33,34,35,36,37,38,39,40,41,42,43]. The included studies involved respondents from 8 countries. The largest number of studies came from the United States and China, each contributing 6 studies (33%). Japan accounted for 2 studies (11%) [37,43]. The United Kingdom [26], Germany [27], and Brazil [35] each contributed 1 study (6%), and one study (6%) was a multicenter survey involving Italy and Belgium [41].

The target populations of the included studies comprised patients, physicians, and caregivers. Studies targeting only patients accounted for 11 studies (61%) [26,27,28,29,30,34,35,36,37,41,42]. Studies targeting only physicians accounted for 2 studies (11%) [31,39]. Additionally, 5 studies (28%) included multiple respondent groups, involving patients and physicians (n = 2, 11%) [33,43], patients and caregivers (n = 2, 11%) [32,38], and patients, physicians, and caregivers (n = 1, 6%) [40].

Across included studies, 11 studies (61%) did not report response rates, and only 2 studies had a response rate higher than 50%. Regarding the survey administration method, 8 studies (44%) collected preference data exclusively online, while 7 studies (39%) used interviewer-administered/in-person methods. Most studies did not explicitly specify the type of treatment assessed (n = 8, 44%) [26,32,33,34,35,36,38,40], but 3 studies (17%) examined preferences for lung cancer chemotherapy [28,31,37], and 2 studies (11%) investigated preferences for traditional Chinese medicine treatments [39,42]. Sample sizes ranged from 24 to 466, with a median of 175. Notably, 13 studies (72%) had sample sizes exceeding 100 [27,28,30,31,33,34,36,37,38,39,40,41,42]. Response rates varied from 5.2% to 99.2%; however, the majority of studies did not explicitly report response rates. Regarding the administration methods of DCE surveys, 9 studies (50%) utilized online surveys [26,29,30,32,36,37,38,41,43], 7 studies (39%) conducted face-to-face interviews [27,28,31,34,39,40,42], and 2 studies (11%) did not explicitly specify the survey administration method [33,35].

### 3.3. Choice Task Design and Analysis

#### 3.3.1. Choice Task Design

Table 2 summarizes the choice task designs in the studies reviewed. All studies reported the process used to determine the attributes and their respective levels, with the literature review being the most commonly used approach. 17 studies (94%) identified attributes and levels through literature reviews [26,27,28,29,30,31,32,33,34,35,36,37,38,39,40,42,43], 11 studies (61%) employed qualitative methods such as respondent interviews and focus groups [27,28,29,32,34,35,36,38,40,41,43], and 9 studies (50%) used expert consultations [26,28,29,31,33,34,35,39,42]. 4 studies (22%) combined all three methods [28,29,34,35]. The number of attributes in the studies typically ranged from 4 to 7, although three studies included 8 [26], 9 [38], and 10 attributes [37], respectively. Each attribute usually had between 2 and 4 levels, with a maximum of 6 levels [43]. The total number of DCE choice sets varied from 13 to 96 [32,40], with 12 studies (67%) using blocking techniques to reduce cognitive burden [27,29,30,33,35,36,37,39,40,41,42,43], randomly assigning respondents to different versions of the choice tasks. More than half of the studies (n = 13, 72%) involved 10 to 14 choice tasks per respondent [26,27,29,30,31,32,33,35,36,37,38,40,41,43], with an average of 12 tasks. However, only 2 studies (11%) included three options per choice task [36,43], offering an opt-out alternative in addition to the two primary options. This indicated that most studies required respondents to choose between two alternatives. The majority of studies (n = 16, 89%) utilized a generic design [26,27,28,29,30,31,32,33,34,35,36,37,39,40,41,42], while only 1 study (6%) implemented a label-based design [43], distinguishing between “Oral treatment” and “Intravenous treatment”. Additionally, 10 studies (56%) conducted pretests with the target population before the formal DCE surveys to assess the validity and clarity of the choice task design and questionnaire [26,28,30,31,33,34,35,39,41,42].

#### 3.3.2. Analysis Procedure

Table 3 summarizes the analytical approaches used in the included studies. Among the econometric models, the mixed logit model was the most frequently applied (n = 10, 56%) [26,28,31,32,33,34,36,39,40,42], followed by the latent class model (n = 4, 22%) [27,30,40,41]. 1 study (6%) employed a mixed multinomial logit model [43], an extension of the multinomial logit model, which accounts for preference heterogeneity among respondents. Regarding variable coding, 6 studies (33%) used categorical variable coding (dummy coding or effect coding) for all attributes [26,27,29,36,37,38], while 11 studies (61%) combined categorical and continuous variable coding [28,30,31,33,34,35,39,40,41,42,43]. Due to the potential confounding between attribute parameter estimates and underlying subjective utility scales, direct comparisons of relative attribute effects based on parameter size and significance are not feasible. Consequently, parameter estimates typically need to be converted into a comparable common scale [44,45]. A total of 15 studies (83%) used a common metric to compare the relative effects of different attributes [26,27,28,29,30,31,32,33,34,35,36,38,40,41,43]. Among these, relative attribute importance (RAI) was the most commonly used metric (n = 8, 44%) [26,28,31,33,38,40,41,43], followed by willingness to pay (WTP) (n = 5, 28%) [28,30,31,34,40], with only a subset of studies incorporating cost variables.

### 3.4. Attribute Classification, Frequency, and Relative Attribute Importance

#### 3.4.1. Attribute Classification

**Figure 2:**
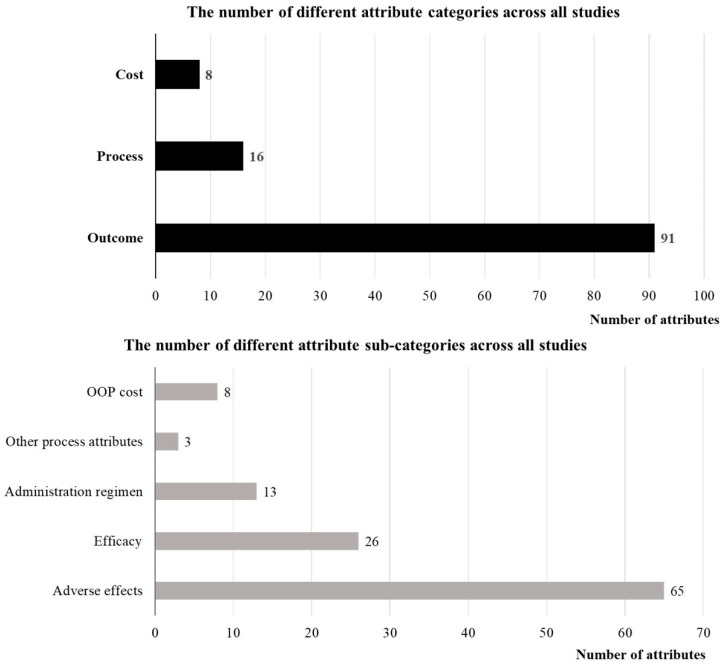
Distribution of the number of different attribute categories and subcategories in all studies. OOP cost, Out-of-pocket cost.

Figure 2 summarizes the classification of attributes across the included studies. To facilitate comparison, attributes were grouped into three main categories: outcome attributes, process attributes, and cost attributes [23,46,47]. Among the 18 studies included, a total of 115 attributes were reported, comprising 91 outcome attributes (79%), 16 process attributes (14%), and 8 cost attributes (7%). Each main category was further divided into distinct subcategories: outcome attributes were classified into treatment efficacy and adverse effects; process attributes included administration regimen (e.g., route of administration, frequency, duration) and other process-related factors; and cost attributes were subdivided into out-of-pocket costs and work-related losses, among others. Within the outcome attributes, 26 attributes (29%) were related to treatment efficacy, while 65 attributes (71%) concerned adverse effects. For process attributes, 13 attributes (81%) were related to the administration regimen, while 3 attributes (19%) fell under other process-related factors. All the attributes in the cost category were related to out-of-pocket costs. A detailed classification of attributes across all studies is provided in Supplementary Materials Table S2.

#### 3.4.2. Attribute Frequency

**Figure 3:**
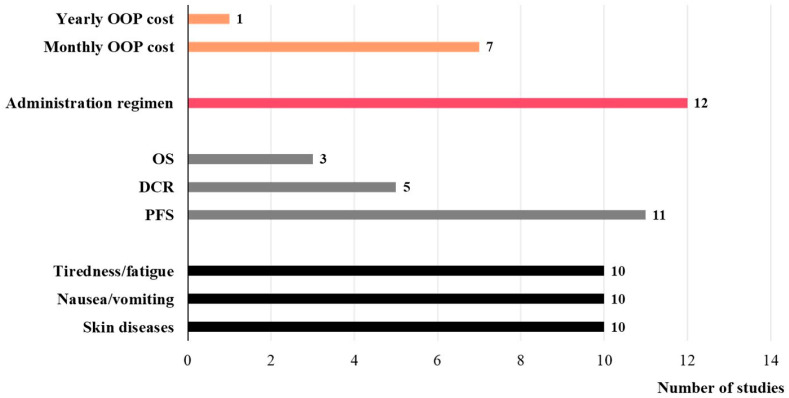
Frequency of occurrence of different attributes in all studies. OOP cost, Out-of-pocket cost; OS, Overall survival; DCR, Disease control rate; PFS, Progression-free survival.

Figure 3 illustrates the frequency of occurrence of each attribute category across all studies. Nearly half of the studies (n = 8, 44%) considered both outcome and process attributes [26,27,29,35,36,37,38,41], while 5 studies (28%) included all three attribute categories [28,30,31,34,43]. 2 studies (11%) focused exclusively on outcome attributes [32,33]. The most common process attribute across all studies was the administration regimen, appearing in 12 studies (67%) [26,27,28,29,31,34,35,36,37,38,41,43]. Among the outcome attributes, “Progression-Free Survival” (PFS) was the most frequently considered attribute in the domain of treatment efficacy, featured in 11 studies (61%) [26,27,28,29,31,32,34,36,37,38,43], followed by “Disease Control Rate” (DCR) (n = 5, 28%) [28,31,34,39,42] and “Overall Survival” (OS) (n = 3, 17%) [37,38,40]. In the domain of adverse effects, the most common attributes were “Skin Diseases”, “Tiredness/Fatigue”, and “Nausea/Vomiting”, each appearing in 10 studies (56%). A total of 8 studies (44%) included cost attributes [28,30,31,34,39,40,42,43], all of which pertained to out-of-pocket treatment expenses, with 7 studies reporting monthly costs and 1 study reporting annual costs.

#### 3.4.3. Relative Attribute Importance

**Figure 4:**
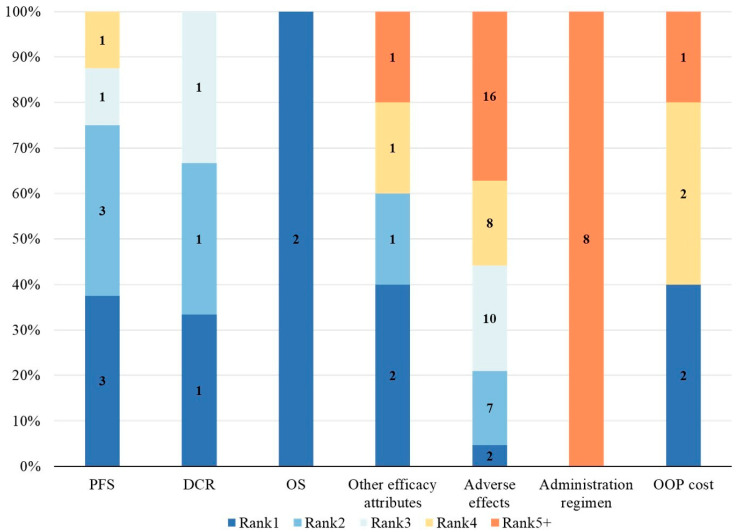
Distribution of rankings for the relative importance of attributes—patients. OOP cost, Out-of-pocket cost; OS, Overall survival; DCR, Disease control rate; PFS, Progression-free survival.

**Figure 5:**
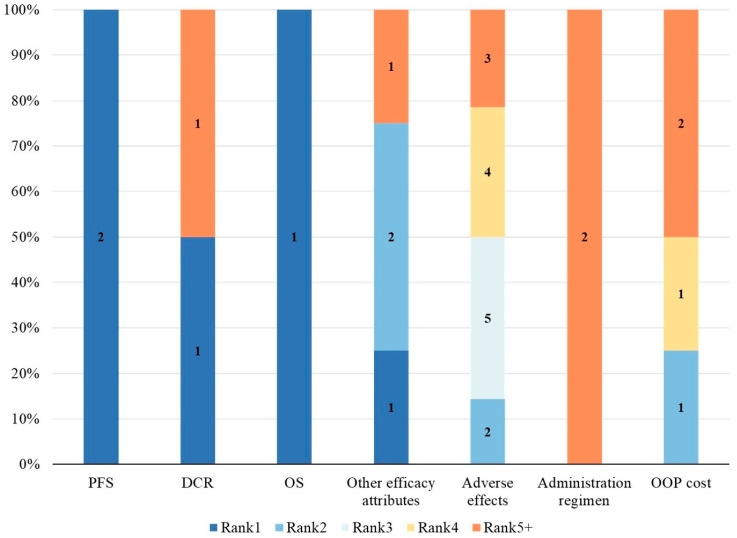
Distribution of rankings for the relative importance of attributes—physicians. OOP cost, Out-of-pocket cost; OS, Overall survival; DCR, Disease control rate; PFS, Progression-free survival.

**Figure 6:**
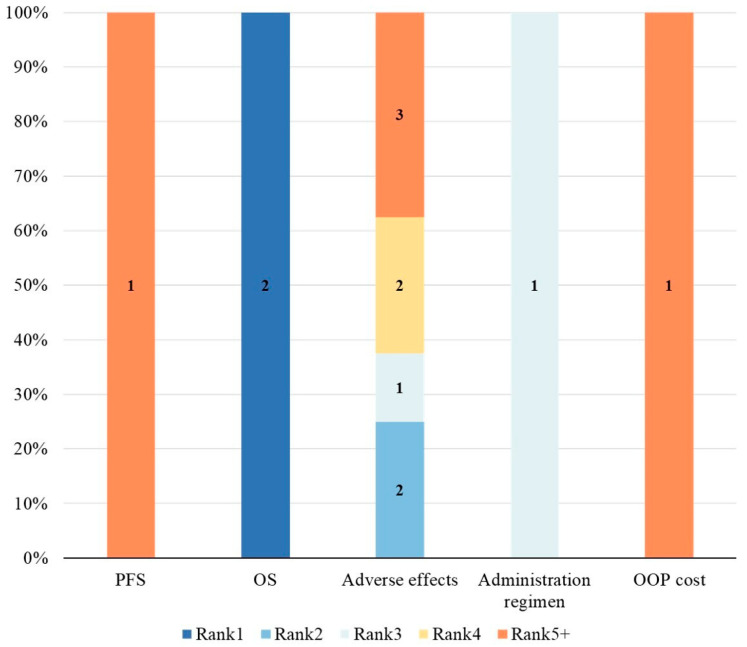
Distribution of rankings for the relative importance of attributes—caregivers. OOP cost, Out-of-pocket cost; OS, Overall survival; PFS, Progression-free survival.

In different studies and stakeholder groups, the relative importance rankings of attributes were not fully consistent. To assess the relative importance of attributes across studies, we utilized Relative Attribute Importance (RAI) or level coefficients. Figure 4, Figure 5 and Figure 6 present the distribution of relative attribute importance rankings in studies reporting patient, physician, and caregiver preferences. Among the studies included in the analysis, over 70% (n = 14) directly reported RAI or provided the level coefficients necessary to compute the relative importance of attributes [26,27,28,29,31,33,34,36,38,39,40,41,42,43]. However, 4 studies were excluded from the analysis due to missing key data or the presence of significant errors in the data, which hindered the calculation of attribute relative importance.

From the patients’ perspective, 10 studies identified outcome attributes as the most important [26,27,29,33,36,38,40,41,42,43], while 2 studies prioritized cost attributes [28,34]. Process attributes were never considered the most important in any of the studies. Among the most important attributes, PFS was the most commonly cited, followed by OS, other efficacy-related attributes, adverse effects, and out-of-pocket costs. Furthermore, the second most important attributes were also predominantly outcome-related. From the physicians’ perspective, all 5 studies considered outcome attributes as the most important [31,33,39,40,43]. Among them, PFS remained the most frequently cited, followed by OS, DCR, and other efficacy-related attributes. The second most important attributes were mainly outcome-related, with only one study categorizing cost attributes as secondary in importance [31]. Studies on caregiver preferences were relatively limited. In the two relevant studies [38,40], OS was considered the most important attribute, followed by adverse effects as the second most important. Out-of-pocket treatment costs were regarded as relatively less important from the caregivers’ perspective.

### 3.5. Quality Assessment

Table 4 presents the quality assessment results of all included studies based on the PREFS checklist. The PREFS scores ranged from 2 to 5 across the 18 studies, with an average score of 3.8. Only 1 study (6%) achieved a perfect score [41]. The majority of studies (n = 13, 72%) received a score of 4 [26,27,28,29,31,32,34,35,36,39,40,42,43], while 3 studies (17%) scored 3 [30,33,37], and 1 study (6%) scored 2 [38]. Specifically, all studies reported the research objectives and outcomes, but only 1 study (6%) reported the differences between responders and non-responders [41]. Although almost all studies provided a detailed and clear explanation of the methods used to assess preferences, one study failed to do so [38]. Additionally, the majority of studies (n = 14, 78%) employed significance tests to evaluate preference outcomes [26,27,28,29,31,32,34,35,36,39,40,41,42,43].

## 4. Discussion

This systematic review identified 18 DCE studies that explored preferences for lung cancer treatment among various stakeholders, including patients, physicians, and caregivers. Lung cancer is one of the most common malignancies globally, and the number of DCE studies on this topic has increased in recent years, further emphasizing the necessity of conducting systematic reviews of DCE studies on preferences for lung cancer treatment. This systematic review summarized the key characteristics of the included studies, including the attributes and levels incorporated, the design of choice tasks, and the relative importance of attributes. The findings of this study will provide valuable insights for future research design, particularly in comparing preferences among different stakeholders, and offer crucial information to support clinical decision-making in lung cancer treatment.

We summarized and categorized the attributes reported in 18 DCE studies, finding that outcome attributes were the most frequently reported, followed by process and cost attributes. Most studies included two types of attributes, while only five incorporated all three. Notably, cost attributes were the least frequently reported, and all studies that included them focused solely on out-of-pocket treatment costs, without addressing other cost-related factors. Compared to outcome and process attributes, cost attributes are often more clearly defined, and their impact is usually perceived more intuitively [47]. However, existing studies primarily focused on out-of-pocket medical costs, neglecting other potential cost factors in lung cancer treatment, such as indirect costs associated with lost productivity due to treatment. Although cost attributes were less frequently studied, their importance in treatment decisions is undeniable. Therefore, it is suggested that future DCE studies incorporate a broader range of cost attributes.

In DCE studies, different groups may choose various preferences in the same scenarios, reflecting their distinct needs and concerns [48,49,50]. Specifically, lung cancer patients tend to focus more on health-related factors. PFS was identified as the most valued attribute by most patients, reflecting lung cancer patients’ desire for disease stability and preservation of quality of life during treatment. Adverse effects were consistently ranked as the least important attribute across many studies, indicating patients’ explicit trade-offs between risks and benefits: to obtain meaningful survival gains, they are willing to accept a certain level of treatment-related risk. However, this is not absolute. Brundage et al. showed that when OS benefits are uncertain, most patients do not prefer treatments that compromise quality of life solely for gains in PFS [51]. In addition, compared with physicians and caregivers, patients placed greater importance on out-of-pocket costs, possibly because they face more immediate concerns about the real-world risk of financial hardship due to illness.

As the primary decision-makers for treatment plans, physicians’ preferences for lung cancer treatment were similar to those of patients, with a greater emphasis on treatment effectiveness. However, physicians exhibit a lower preference for out-of-pocket costs. This may stem from their disease-centered approach to medical decision-making, where survival benefit remains the primary objective, while other attributes are viewed as secondary factors subject to trade-offs. For caregivers, their main concern is the patient’s OS. Extending a patient’s lifespan is not only a medical objective but also reflects the emotional bond with the patient and the value of their caregiving work. However, compared to physicians and patients, caregivers do not prioritize PFS or adverse effects. This may be because PFS benefits and adverse effects do not directly impact their caregiving burden and are difficult to translate into tangible, daily value.

In summary, although outcome attributes were generally regarded as the most important by different stakeholders, the degree of importance attached to specific outcome attributes varied. It should be noted that the relative importance of outcome, process, and cost attributes may also be context-dependent. Differences in the healthcare system and risk tolerance may partly shape how stakeholders trade off survival benefits, adverse effects, and costs. Furthermore, among DCE studies involving multiple groups, only one study varied the attributes used between two respondent groups. Although most DCE studies on lung cancer treatment preferences involved similar attributes, the concerns of different groups were not identical. To better reflect the true preferences of different stakeholders, future research should focus on tailoring attributes and level selection to the features of target populations, thereby enhancing the practical applicability of the findings.

In DCE designs, respondents are typically required to make decisions from a set of predefined choice tasks. However, we observed that most of the included studies did not include “opt-out” or “status quo” options in their choice task designs, instead requiring respondents to choose between two alternatives. While this approach may reduce the cognitive burden on respondents, it does not fully reflect real-world situations where respondents may decline all treatment options or prefer to maintain current treatment due to uncertainties or other factors. Including “opt-out” or “status quo” options would more accurately reflect respondents’ decision-making behaviors in such a context [52]. Additionally, forced choices may introduce bias by failing to capture respondents’ true preferences [53,54]. This may overstate respondents’ willingness to trade off treatment benefits against harms or costs. While “opt-out” or “status quo” options are common and important in real-world decisions, they have received limited attention in DCE studies. Therefore, we believe it is feasible that future DCE studies incorporate “opt-out” or “status quo” options into their design to simulate a more realistic choice environment. At the same time, allowing an “opt-out” or “status quo” option may increase task complexity or lead to higher opt-out rates, and these trade-offs should be considered when designing future studies.

The quality of the included studies was assessed using the PREFS checklist. Overall, the studies demonstrated high quality, with 72% scoring 4 out of 5 on the checklist. The main reason for score deductions was related to the “respondent” dimension, which assesses the risk of selection bias [19]. Selection bias may arise if responders differ from non-responders. Only one study reported similarities between responders and non-responders, while the others did not report such information, which could lead to non-response bias. Therefore, although the overall quality of these DCE studies is relatively high, certain areas still need improvement to enhance the representativeness of the results.

Although this review closely adhered to established best practice guidelines for systematic reviews, several potential limitations remain. First, the number of DCE studies included was limited, especially those involving physicians and caregivers. A total of 18 studies were included in this review, of which only 5 involved physicians and 3 involved caregivers. This may affect the generalizability of the results, particularly when exploring the preference differences among various stakeholders. Second, some studies did not report estimates of attribute preference weights, which prevented their inclusion in the analysis of relative attribute importance. Third, this review did not include a comparison of WTP among patients, physicians, and caregivers. While WTP is an important economic indicator, it was reported for caregivers in only one study, which resulted in an insufficient sample size for a representative comparison. Fourth, this review did not include grey literature, which may increase the risk of publication bias. Fifth, this review utilized the PREFS checklist to evaluate the quality of DCE studies, which focuses on five key areas and cannot cover all critical aspects of DCE research, such as the attribute identification and selection process or the use of econometric models. Finally, this review covered publications up to 12 July 2024, and more recent studies may not have been captured.

## 5. Conclusions

This systematic review synthesized evidence on the treatment preferences of patients, physicians, and caregivers for lung cancer. Existing studies have differences in the coverage of study populations, the scope of attributes, and the research design of experiments. Most DCEs have focused on patients’ preferences for treatment efficacy and adverse effects, with less attention to other attributes that may influence treatment choices. Moreover, there is heterogeneity in the preferences for lung cancer treatment among physicians, patients, and caregivers. Therefore, to better understand and reach consensus on lung cancer treatment among stakeholders, more DCE or other explicit preference studies are needed to provide evidence-based support for personalized lung cancer treatment plans.

## Figures and Tables

**Table 1:** Characteristics of included studies.

First Author, Year of Publication	Country	Disease Conditions	Modalities of Treatment	Target Group	Sample Size	Response Rate	Survey Administration Method
Bridges et al., 2012 [26]	UK	NSCLC	Not stated	Patient	89	20.3%	Online survey
Mühlbacher et al., 2015 [27]	Germany	Stage IV NSCLC	Drug therapy	Patient	211	Not stated	Interviewer administered/in person
Sun et al., 2019 [28]	China	NSCLC	Chemotherapy	Patient	361	Not stated	Interviewer administered/in person
Bridges et al., 2019 [29]	USA	EGFRm NSCLC	Targeted therapy	Patient	90	Not stated	Online survey
MacEwan et al., 2020 [30]	USA	Stage III/IV NSCLC	First-line treatment	Patient	199	Not stated	Online survey
Sun et al., 2020 [31]	China	NSCLC	Chemotherapy	Physician	184	Not stated	Interviewer administered/in person
Janssen et al., 2020 [32]	USA	LC	Not stated	Patient	87	44%	Questionnaire/Online survey
				Caregiver	24	24%	
Hauber et al., 2020 [33]	USA	Advanced NSCLC	Not stated	Patient	200	5.2%	Not stated
				Physician	102	10.1%	
Liu et al., 2021 [34]	China	NSCLC	Not stated	Patient	202	67.3%	Interviewer administered/in person
Meirelles et al., 2021 [35]	Brazil	Locally advanced, metastatic or recurrent NSCLC	Not stated	Patient	65	Not stated	Not stated
Janse et al., 2021 [36]	USA	NSCLC	Not stated	Patient	466	Not stated	Online survey
Sugitani et al., 2021 [37]	Japan	LC	Chemotherapy	Patient	191	44.6%	Online survey
Yong et al., 2022 [38]	USA	Metastatic NSCLC	Not stated	Patient	308	Not stated	Online survey
				Caregiver	166	Not stated	
Yan et al., 2022 [39]	China	LC	Traditional Chinese medicine treatment	Physician	185	Not stated	Interviewer administered/in person
Zhang et al., 2022 [40]	China	LC	Not stated	Patient	161	87.5%	Interviewer administered/in person
				Physician	121	99.2%	
				Caregiver	161	87.5%	
Oliveri et al., 2023 [41]	Italy and Belgium	NSCLC	Immunotherapy	Patient	307	71.6%	Online survey
Teng et al., 2023 [42]	China	LC	Traditional Chinese medicine treatment	Patient	347	Not stated	Interviewer administered/in person
Hata et al., 2024 [43]	Japan	EGFRm NSCLC	Novel treatments	Patient	54	Not stated	Online survey
				Physician	74	Not stated	

Note: LC, Lung cancer; NSCLC, Non-small-cell lung cancer; EGFRm, Epidermal growth factor receptor mutation.

**Table 2:** Choice task design of included studies.

Category	n (%) (N = 18)
**Selection Of Attributes**	
Literature review	17 (94%)
Qualitative work	11 (61%)
Expert consultation	9 (50%)
All 3 methods	4 (22%)
**Number of attributes**	
4	2 (11%)
5	2 (11%)
6	7 (39%)
7	4 (22%)
>7	3 (17%)
**Mean levels per attribute**	
2–3	7 (39%)
3–4	10 (56%)
4–5	1 (6%)
**Design software**	
SAS	6 (33%)
Ngene	6 (33%)
Sawtooth	2 (11%)
R	1 (6%)
Not clearly reported	3 (17%)
**Number of choices sets**	
<24	6 (33%)
24–48	8 (44%)
>48	3 (17%)
Not clearly reported	1 (6%)
**Block**	
Yes	12 (67%)
No	5 (28%)
Not clearly reported	1 (6%)
**Number of choices per respondent**	
<10	2 (11%)
10–14	13 (72%)
15–18	3 (17%)
**Number of alternatives**	
2	16 (89%)
3	2 (11%)
**Opt-out or status quo options**	
Yes	2 (11%)
No	16 (89%)
**Label design**	
Yes	1 (6%)
No	16 (89%)
Not clearly reported	1 (6%)
**Piloting**	
Yes	10 (56%)
No	8 (44%)

**Table 3:** Analysis procedure of included studies.

Category	n (%) (N = 18)
**Econometric model**	
Mixed logit model	10 (56%)
Latent class model	4 (22%)
Conditional logit model	3 (17%)
Hierarchical Bayesian model	2 (11%)
Mixed multinomial logit model	1 (6%)
**Coding type**	
Effect coding	9 (50%)
Dummy coding	8 (44%)
Continuous	12 (67%)
**Analysis software**	
Stata	9 (50%)
R	3 (17%)
Nlogit	2 (11%)
Sawtooth	1 (6%)
Not clearly reported	4 (22%)
**Common metric to compare relative attribute effects**	
Relative attribute importance	8 (44%)
Willingness to pay	5 (28%)
Progression-free survival equivalents	4 (22%)
Maximum acceptable risk	3 (17%)
Minimum acceptable benefit	2 (11%)
Probability analysis	1 (6%)
Not clearly reported	3 (17%)

Note: Studies may report more than one option; the percentages and counts may not sum to 100% or N = 18.

**Table 4:** Results of the quality assessment of the included studies according to the PREFS checklist.

Study	Purpose	Respondents	Explanation	Findings	Significance	Score
Bridges et al., 2012 [26]	1	0	1	1	1	4
Mühlbacher et al., 2015 [27]	1	0	1	1	1	4
Sun et al., 2019 [28]	1	0	1	1	1	4
Bridges et al., 2019 [29]	1	0	1	1	1	4
MacEwan et al., 2020 [30]	1	0	1	1	0	3
Sun et al., 2020 [31]	1	0	1	1	1	4
Janssen et al., 2020 [32]	1	0	1	1	1	4
Hauber et al., 2020 [33]	1	0	1	1	0	3
Liu et al., 2021 [34]	1	0	1	1	1	4
Meirelles et al., 2021 [35]	1	0	1	1	1	4
Janse et al., 2021 [36]	1	0	1	1	1	4
Sugitani et al., 2021 [37]	1	0	1	1	0	3
Yong et al., 2022 [38]	1	0	0	1	0	2
Yan et al., 2022 [39]	1	0	1	1	1	4
Zhang et al., 2022 [40]	1	0	1	1	1	4
Oliveri et al., 2023 [41]	1	1	1	1	1	5
Teng et al., 2023 [42]	1	0	1	1	1	4
Hata et al., 2024 [43]	1	0	1	1	1	4

## Data Availability

No new data were created or analyzed in this study. Data sharing is not applicable to this article.

## Supplementary Materials

The following supporting information can be downloaded at https://www.mdpi.com/article/10.3390/healthcare14050584/s1, Table S1: Search strategy; Table S2: Attributes included in all studies and their classification.

## References

[1] Bray F., Laversanne M., Sung H., Ferlay J., Siegel R.L., Soerjomataram I., Jemal A. (2024). Global cancer statistics 2022: GLOBOCAN estimates of incidence and mortality worldwide for 36 cancers in 185 countries. CA A Cancer J. Clin..

[2] Ferlay J., Ervik M., Lam F., Laversanne M., Colombet M., Mery L., Piñeros M., Znaor A., Soerjomataram I., Bray F. (2024). Global Cancer Observatory: Cancer Today (Version 1.1).

[3] Ramalingam S.S., Owonikoko T.K., Khuri F.R. (2011). Lung cancer: New biological insights and recent therapeutic advances. CA A Cancer J. Clin..

[4] Arbour K.C., Riely G.J. (2019). Systemic Therapy for Locally Advanced and Metastatic Non-Small Cell Lung Cancer: A Review. JAMA.

[5] Li Y., Yan B., He S. (2023). Advances and challenges in the treatment of lung cancer. Biomed. Pharmacother..

[6] Miao D., Zhao J., Han Y., Zhou J., Li X., Zhang T., Li W., Xia Y. (2024). Management of locally advanced non-small cell lung cancer: State of the art and future directions. Cancer Commun..

[7] Meyer M.L., Fitzgerald B.G., Paz-Ares L., Cappuzzo F., Jänne P.A., Peters S., Hirsch F.R. (2024). New promises and challenges in the treatment of advanced non-small-cell lung cancer. Lancet.

[8] Mark T.L., Swait J. (2004). Using stated preference and revealed preference modeling to evaluate prescribing decisions. Health Econ..

[9] Bridges J.F., Hauber A.B., Marshall D., Lloyd A., Prosser L.A., Regier D.A., Johnson F.R., Mauskopf J. (2011). Conjoint analysis applications in health--a checklist: A report of the ISPOR Good Research Practices for Conjoint Analysis Task Force. Value Health J. Int. Soc. Pharmacoecon. Outcomes Res..

[10] Lancsar E., Louviere J. (2008). Conducting discrete choice experiments to inform healthcare decision making: A user’s guide. PharmacoEconomics.

[11] Clark M.D., Determann D., Petrou S., Moro D., de Bekker-Grob E.W. (2014). Discrete choice experiments in health economics: A review of the literature. PharmacoEconomics.

[12] Soekhai V., de Bekker-Grob E.W., Ellis A.R., Vass C.M. (2019). Discrete Choice Experiments in Health Economics: Past, Present and Future. PharmacoEconomics.

[13] Hauber A.B., González J.M., Groothuis-Oudshoorn C.G., Prior T., Marshall D.A., Cunningham C., IJzerman M.J., Bridges J.F. (2016). Statistical Methods for the Analysis of Discrete Choice Experiments: A Report of the ISPOR Conjoint Analysis Good Research Practices Task Force. Value Health J. Int. Soc. Pharmacoecon. Outcomes Res..

[14] Lancsar E., Fiebig D.G., Hole A.R. (2017). Discrete Choice Experiments: A Guide to Model Specification, Estimation and Software. PharmacoEconomics.

[15] Schmidt K., Damm K., Prenzler A., Golpon H., Welte T. (2016). Preferences of lung cancer patients for treatment and decision-making: A systematic literature review. Eur. J. Cancer Care.

[16] Sugitani Y., Sugitani N., Ono S. (2020). Quantitative Preferences for Lung Cancer Treatment from the Patients’ Perspective: A Systematic Review. Patient.

[17] Moher D., Liberati A., Tetzlaff J., Altman D.G., PRISMA Group (2009). Preferred reporting items for systematic reviews and meta-analyses: The PRISMA statement. PLoS Med..

[18] De Bekker-Grob E.W., Ryan M., Gerard K. (2012). Discrete choice experiments in health economics: A review of the literature. Health Econ..

[19] Joy S.M., Little E., Maruthur N.M., Purnell T.S., Bridges J.F. (2013). Patient preferences for the treatment of type 2 diabetes: A scoping review. PharmacoEconomics.

[20] Showalter T.N., Mishra M.V., Bridges J.F. (2015). Factors that influence patient preferences for prostate cancer management options: A systematic review. Patient Prefer. Adherence.

[21] Zhou M., Thayer W.M., Bridges J.F.P. (2018). Using Latent Class Analysis to Model Preference Heterogeneity in Health: A Systematic Review. PharmacoEconomics.

[22] Lack A., Hiligsmann M., Bloem P., Tünneßen M., Hutubessy R. (2020). Parent, provider and vaccinee preferences for HPV vaccination: A systematic review of discrete choice experiments. Vaccine.

[23] Jiang S., Ren R., Gu Y., Jeet V., Liu P., Li S. (2023). Patient Preferences in Targeted Pharmacotherapy for Cancers: A Systematic Review of Discrete Choice Experiments. PharmacoEconomics.

[24] Baird T.A., Wright D.R., Britto M.T., Lipstein E.A., Trout A.T., Hayatghaibi S.E. (2023). Patient Preferences in Diagnostic Imaging: A Scoping Review. Patient.

[25] Hinzpeter E.L., Nagendra L., Kairies-Schwarz N., Beaudart C., Hiligsmann M. (2024). Stated Preferences of At-Risk Populations for the Treatment of Osteoporosis: A Systematic Review. Patient.

[26] Bridges J.F., Mohamed A.F., Finnern H.W., Woehl A., Hauber A.B. (2012). Patients’ preferences for treatment outcomes for advanced non-small cell lung cancer: A conjoint analysis. Lung Cancer.

[27] Mühlbacher A.C., Bethge S. (2015). Patients’ preferences: A discrete-choice experiment for treatment of non-small-cell lung cancer. Eur. J. Health Econ. HEPAC Health Econ. Prev. Care.

[28] Sun H., Wang H., Xu N., Li J., Shi J., Zhou N., Ni M., Hu X., Chen Y. (2019). Patient Preferences For Chemotherapy In The Treatment Of Non-Small Cell Lung Cancer: A Multicenter Discrete Choice Experiment (DCE) Study In China. Patient Prefer. Adherence.

[29] Bridges J.F., la Cruz M., Pavilack M., Flood E., Janssen E.M., Chehab N., Fernandes A.W. (2019). Patient preferences for attributes of tyrosine kinase inhibitor treatments for EGFR mutation-positive non-small-cell lung cancer. Future Oncol..

[30] MacEwan J.P., Gupte-Singh K., Zhao L.M., Reckamp K.L. (2020). Non-Small Cell Lung Cancer Patient Preferences for First-Line Treatment: A Discrete Choice Experiment. MDM Policy Pract..

[31] Sun H., Wang H., Shi L., Wang M., Li J., Shi J., Ni M., Hu X., Chen Y. (2020). Physician preferences for chemotherapy in the treatment of non-small cell lung cancer in China: Evidence from multicentre discrete choice experiments. BMJ Open.

[32] Janssen E.M., Dy S.M., Meara A.S., Kneuertz P.J., Presley C.J., Bridges J.F.P. (2020). Analysis of Patient Preferences in Lung Cancer—Estimating Acceptable Tradeoffs Between Treatment Benefit and Side Effects. Patient Prefer. Adherence.

[33] Hauber B., Penrod J.R., Gebben D., Musallam L. (2020). The Value of Hope: Patients’ and Physicians’ Preferences for Survival in Advanced Non-Small Cell Lung Cancer. Patient Prefer. Adherence.

[34] Liu F., Hu H., Wang J., Chen Y., Hui S., Hu M. (2021). A Study of Patient Preferences for the Treatment of Non-small Cell Lung Cancer in Western China: A Discrete-Choice Experiment. Front. Public Health.

[35] Meirelles I., Magliano C. (2021). Stated Preferences in Non-Small-Cell Lung Cancer: A Discrete Choice Experiment. Patient Prefer. Adherence.

[36] Janse S., Janssen E., Huwig T., Basu Roy U., Ferris A., Presley C.J., Bridges J.F.P. (2021). Line of therapy and patient preferences regarding lung cancer treatment: A discrete-choice experiment. Curr. Med. Res. Opin..

[37] Sugitani Y., Ito K., Ono S. (2021). Patient Preferences for Attributes of Chemotherapy for Lung Cancer: Discrete Choice Experiment Study in Japan. Front. Pharmacol..

[38] Yong C., Cambron-Mellott M.J., Seal B., Will O., Maculaitis M.C., Clapp K., Mulvihill E., Cotarla I., Mehra R. (2022). Patient and Caregiver Preferences for First-Line Treatments of Metastatic Non-Small Cell Lung Cancer: A Discrete Choice Experiment. Patient Prefer. Adherence.

[39] Yan J., Wei Y., Teng Y., Liu S., Li F., Bao S., Ren Y., Chen Y. (2022). Physician Preferences and Shared-Decision Making for the Traditional Chinese Medicine Treatment of Lung Cancer: A Discrete-Choice Experiment Study in China. Patient Prefer. Adherence.

[40] Zhang M., He X., Wu J., Wang X., Jiang Q., Xie F. (2022). How Do Treatment Preferences of Patients With Cancer Compare With Those of Oncologists and Family Members? Evidence From a Discrete Choice Experiment in China. Value Health J. Int. Soc. Pharmacoecon. Outcomes Res..

[41] Oliveri S., Lanzoni L., Veldwijk J., de Wit G.A., Petrocchi S., Janssens R., Schoefs E., Smith M.Y., Smith I., Nackaerts K. (2023). Balancing benefits and risks in lung cancer therapies: Patient preferences for lung cancer treatment alternatives. Front. Psychol..

[42] Teng Y., Wei Y., Chen Y., Yan J., Liu S., Li F., Bao S., Ren Y., Liu L., Yang Y. (2023). Patient preferences and shared decision making for the traditional Chinese medicine treatment of lung cancer: A discrete choice experiment study. Integr. Med. Res..

[43] Hata A., Fifer S., Hasegawa K., Ando E., Kasahara-Kiritani M., Takahashi M., Ordman R., Toh L., Inoue A. (2024). Treatment preferences among Japanese patients and physicians for epidermal growth factor receptor-mutant non-small cell lung cancer. Cancer Med..

[44] Lancsar E., Louviere J., Flynn T. (2007). Several methods to investigate relative attribute impact in stated preference experiments. Soc. Sci. Med..

[45] Mandeville K.L., Lagarde M., Hanson K. (2014). The use of discrete choice experiments to inform health workforce policy: A systematic review. BMC Health Serv. Res..

[46] Bien D.R., Danner M., Vennedey V., Civello D., Evers S.M., Hiligsmann M. (2017). Patients’ Preferences for Outcome, Process and Cost Attributes in Cancer Treatment: A Systematic Review of Discrete Choice Experiments. Patient.

[47] Tünneßen M., Hiligsmann M., Stock S., Vennedey V. (2020). Patients’ preferences for the treatment of anxiety and depressive disorders: A systematic review of discrete choice experiments. J. Med. Econ..

[48] Montgomery A.A., Fahey T. (2001). How do patients’ treatment preferences compare with those of clinicians?. Qual. Health Care QHC.

[49] Mühlbacher A.C., Juhnke C. (2013). Patient preferences versus physicians’ judgement: Does it make a difference in healthcare decision making?. Appl. Health Econ. Health Policy.

[50] Harrison M., Milbers K., Hudson M., Bansback N. (2017). Do patients and health care providers have discordant preferences about which aspects of treatments matter most? Evidence from a systematic review of discrete choice experiments. BMJ Open.

[51] Brundage M.D., Booth C.M., Eisenhauer E.A., Galica J., Kankesan J., Karim S., Koven R., McDonald V., Ng T., O’Donnell J. (2023). Patients’ attitudes and preferences toward delayed disease progression in the absence of improved survival. J. Natl. Cancer Inst..

[52] Campbell D., Erdem S. (2019). Including Opt-Out Options in Discrete Choice Experiments: Issues to Consider. Patient.

[53] Veldwijk J., Lambooij M.S., de Bekker-Grob E.W., Smit H.A., de Wit G.A. (2014). The effect of including an opt-out option in discrete choice experiments. PLoS ONE.

[54] Train K.E. (2009). Discrete Choice Methods with Simulation.

