# Supplementary material for: Patient, Physician, and Caregiver Preferences for Lung Cancer Treatment: A Systematic Review of Discrete Choice Experiments
Source: Healthcare (Basel). 2026 Feb 26;14(5):584. doi: 10.3390/healthcare14050584 (PMC12984755; doi:10.3390/healthcare14050584)
Supplement: Supplementary file 1 [file healthcare-14-00584-s001.zip › healthcare-4060749-supplementary.pdf]

Supplementary Table S1. Search strategy

Database	Search term
**PubMed**	("discrete choice" OR "choice experiment*" OR "choice model*" OR DCE OR "conjoint experiment*" OR "conjoint analysis" OR “conjoint measurement*” OR "conjoint stud*" OR "choice? based conjoint" OR "pairwise choice*" OR "paired comparison*" OR "part-worth utilit*" OR "stated choice*" OR "stated preference*" OR "factorial survey*" OR “functional measurement*") AND (“Lung Neoplasms"[Mesh] OR “lung cancer*”)
**Web of science**	TS=("discrete choice" OR "choice experiment*" OR "choice model*" OR DCE OR "conjoint experiment*" OR "conjoint analysis" OR “conjoint measurement*” OR "conjoint stud*" OR "choice? based conjoint" OR "pairwise choice*" OR "paired comparison*" OR "part-worth utilit*" OR "stated choice*" OR "stated preference*" OR "factorial survey*" OR “functional measurement*") AND TS=(“Lung Neoplasms" OR “lung cancer*”) AND DT==("ARTICLE")
**Embase**	('discrete choice' OR 'choice experiment*' OR 'choice model*' OR dce OR 'conjoint experiment*' OR 'conjoint analysis'/exp OR 'conjoint analysis' OR 'conjoint measurement*' OR 'conjoint stud*' OR 'choice? based conjoint' OR 'pairwise choice*' OR 'paired comparison*' OR 'part-worth utilit*' OR 'stated choice*' OR 'stated preference*' OR 'factorial survey*' OR 'functional measurement*') AND ('lung neoplasms' OR 'lung cancer*') AND 'article'/it
**Scopus**	TITLE-ABS-KEY ("discrete choice" OR "choice experiment*" OR "choice model*" OR dce OR "conjoint experiment*" OR "conjoint analysis" OR "conjoint measurement*" OR "conjoint stud*" OR "choice? based conjoint" OR "pairwise choice*" OR "paired comparison*" OR "part-worth utilit*" OR "stated choice*" OR "stated preference*" OR "factorial survey*" OR "functional measurement*") AND TITLE-ABS-KEY ("Lung Neoplasms" OR "lung cancer*") AND (LIMIT-TO (DOCTYPE , "ar"))

Supplementary Table S2.Attributes included in all studies and their classification

Study	Attribute	Classification	
Bridges et al., 2012	Progression-free survival	Outcome	Efficacy
	Pain, coughing, and shortness of breath	Outcome	Adverse effects
	Rash	Outcome	Adverse effects
	Diarrhoea	Outcome	Adverse effects
	Fatigue (tiredness)	Outcome	Adverse effects
	Nausea and vomiting	Outcome	Adverse effects
	Fever and infection	Outcome	Adverse effects
	Oral or infusion	Process	Administration regimen
Mühlbacher et al., 2015	Time without tumor progression	Outcome	Efficacy
	Side effect of skin	Outcome	Adverse effects
	Nausea and vomiting	Outcome	Adverse effects
	Diarrhea	Outcome	Adverse effects
	Tiredness/Fatigue	Outcome	Adverse effects
	Tumor-related symptoms	Outcome	Adverse effects
	Mode of administration	Process	Administration regimen
Sun et al., 2019	Progression free survival	Outcome	Efficacy
	Disease control rate	Outcome	Efficacy
	Rash	Outcome	Adverse effects
	Nausea and vomiting	Outcome	Adverse effects
	Tiredness	Outcome	Adverse effects
	Out-of-pocket costs	Cost	OOP cost
	Administration mode	Process	Administration regimen
Bridges et al., 2019	Progression-free survival	Outcome	Efficacy
	Rash	Outcome	Adverse effects
	Diarrhea	Outcome	Adverse effects
	Nausea and vomiting	Outcome	Adverse effects
	Fatigue	Outcome	Adverse effects
	Mode of administration	Process	Administration regimen
MacEwan et al., 2020	Two-year survival rate	Outcome	Efficacy
	Out of pocket costs	Cost	OOP cost
	Adverse events	Outcome	Adverse effects
	Mechanism of action	Process	Other process attribute
	Subsequent treatment options	Process	Other process attribute
	Genetic testing treatment delay	Process	Other process attribute
Sun et al., 2020	Progression-free survival	Outcome	Efficacy
	Disease control rate	Outcome	Efficacy
	Risk of moderate side effects	Outcome	Adverse effects
	Risk of severe side effects	Outcome	Adverse effects
	Cost	Cost	OOP cost
	Mode of administration	Process	Administration regimen
Janssen et al., 2020	Progression free survival	Outcome	Efficacy
	Short-term side effects	Outcome	Adverse effects

	Physical long-term effects	Outcome	Adverse effects
	Emotional long-term effects	Outcome	Adverse effects
	Cognitive long-term effects	Outcome	Adverse effects
	Functional long-term effects	Outcome	Adverse effects
Hauber et al., 2020	Expected survival	Outcome	Efficacy
	Best-case survival	Outcome	Efficacy
	Worst-case survival	Outcome	Efficacy
	Fatigue	Outcome	Adverse effects
	Nausea	Outcome	Adverse effects
	Risk of febrile neutropenia (fever)	Outcome	Adverse effects
Liu et al., 2021	Progression-free survival	Outcome	Efficacy
	Disease control rate	Outcome	Efficacy
	Rash	Outcome	Adverse effects
	Nausea and vomiting	Outcome	Adverse effects
	Weakness and fatigue	Outcome	Adverse effects
	Cost	Cost	OOP cost
	Mode of administration	Process	Administration regimen
Meirelles et al., 2021	Tiredness	Outcome	Adverse effects
	Hair loss	Outcome	Adverse effects
	Skin rash	Outcome	Adverse effects
	Hospitalization	Outcome	Adverse effects
	Administration mode	Process	Administration regimen
	Survival	Outcome	Efficacy
Janse et al., 2021	Progression free survival	Outcome	Efficacy
	Severity of short-term side effects	Outcome	Adverse effects
	Severity of long-term side effects	Outcome	Adverse effects
	Risk of late-onset side effects	Outcome	Adverse effects
	Mode of administration	Process	Administration regimen
Sugitani et al., 2021	OS	Outcome	Efficacy
	PFS	Outcome	Efficacy
	Diarrhea	Outcome	Adverse effects
	Nausea or vomiting	Outcome	Adverse effects
	Fatigue or general malaise	Outcome	Adverse effects
	Rash	Outcome	Adverse effects
	Interstitial lung disease	Outcome	Adverse effects
	Bone marrow suppression	Outcome	Adverse effects
	Frequency of administration	Process	Administration regimen
	Duration of administration	Process	Administration regimen
Yong et al., 2022	Median OS	Outcome	Efficacy
	Median PFS	Outcome	Efficacy
	Nausea (all grades)	Outcome	Adverse effects
	Neuropathy (all grades)	Outcome	Adverse effects
	Pneumonitis (all grades)	Outcome	Adverse effects
	Fatigue (grade 3/4)	Outcome	Adverse effects

	Skin rash (all grades)	Outcome	Adverse effects
	Serious (grade 3/4) AE	Outcome	Adverse effects
	Regimen	Process	Administration regimen
Yan et al., 2022	Disease control rate	Outcome	Efficacy
	Nausea & vomiting	Outcome	Adverse effects
	Risk of side effects	Outcome	Adverse effects
	Out-of-pocket costs	Cost	OOP cost
Zhang et al., 2022	Overall survival time	Outcome	Efficacy
	Risk of severe adverse effect	Outcome	Adverse effects
	Severity of pain	Outcome	Adverse effects
	Appetite	Outcome	Adverse effects
	Physical functioning status	Outcome	Adverse effects
	Monthly treatment cost	Cost	OOP cost
Oliveri et al., 2023	How the treatment is given to you	Process	Administration regimen
	Chance of surviving 5 years after starting this cancer treatment	Outcome	Efficacy
	Chance of long lasting skin problems	Outcome	Adverse effects
	Chance of being extremely tired	Outcome	Adverse effects
	Severity of hair loss	Outcome	Adverse effects
Teng et al., 2023	Disease control rate	Outcome	Efficacy
	Nausea & vomiting	Outcome	Adverse effects
	Risk of side effects	Outcome	Adverse effects
	Out-of-pocket costs	Cost	OOP cost
Hata et al., 2024	Frequency of administration	Process	Administration regimen
	Overall response rate (ORR)	Outcome	Efficacy
	Average progression-free survival (PFS)	Outcome	Efficacy
	Chance of experiencing mild – moderate gastrointestinal side effects	Outcome	Adverse effects
	Chance of experiencing mild – moderate skin-related side effects	Outcome	Adverse effects
	Chance of experiencing any severe side effects	Outcome	Adverse effects
	Yearly cost	Cost	OOP cost
